# Development of cassava common mosaic virus-based vector for protein expression and gene editing in cassava

**DOI:** 10.1186/s13007-023-01055-5

**Published:** 2023-08-03

**Authors:** Decai Tuo, Yuan Yao, Pu Yan, Xin Chen, Feihong Qu, Weiqian Xue, Jinping Liu, Hua Kong, Jianchun Guo, Hongguang Cui, Zhaoji Dai, Wentao Shen

**Affiliations:** 1 National Key Laboratory for Tropical Crops Breeding, Key Laboratory of Biology and Genetic Resources of Tropical Crops (Ministry of Agriculture and Rural Affairs), Hainan Key Laboratory for Protection and Utilization of Tropical Bioresources, Institute of Tropical Bioscience and Biotechnology, Sanya Research Institute, Hainan Institute for Tropical Agricultural Resources, Chinese Academy of Tropical Agricultural Sciences, Haikou & Sanya, Hainan, China; 2 School of Tropical Agriculture and Forestry, Sanya Nanfan Research Institute, Hainan University, Haikou & Sanya, Hainan, China

**Keywords:** Cassava, Cassava common mosaic virus, Protein overexpression, Genome editing

## Abstract

**Background:**

Plant virus vectors designed for virus-mediated protein overexpression (VOX), virus-induced gene silencing (VIGS), and genome editing (VIGE) provide rapid and cost-effective tools for functional genomics studies, biotechnology applications and genome modification in plants. We previously reported that a cassava common mosaic virus (CsCMV, genus *Potexvirus*)-based VIGS vector was used for rapid gene function analysis in cassava. However, there are no VOX and VIGE vectors available in cassava.

**Results:**

In this study, we developed an efficient VOX vector (CsCMV2-NC) for cassava by modifying the CsCMV-based VIGS vector. Specifically, the length of the duplicated putative subgenomic promoter (SGP1) of the CsCMV *CP* gene was increased to improve heterologous protein expression in cassava plants. The modified CsCMV2-NC-based VOX vector was engineered to express genes encoding green fluorescent protein (GFP), bacterial phytoene synthase (crtB), and *Xanthomonas axonopodis* pv. *manihotis* (*Xam*) type III effector XopAO1 for viral infection tracking, carotenoid biofortification and *Xam* virulence effector identification in cassava. In addition, we used CsCMV2-NC to deliver single guide RNAs (gMePDS1/2) targeting two loci of the cassava phytoene desaturase gene (*MePDS*) in Cas9-overexpressing transgenic cassava lines. The CsCMV-gMePDS1/2 efficiently induced deletion mutations of the targeted *MePDS* with the albino phenotypes in systemically infected cassava leaves.

**Conclusions:**

Our results provide a useful tool for rapid and efficient heterologous protein expression and guide RNA delivery in cassava. This expands the potential applications of CsCMV-based vector in gene function studies, biotechnology research, and precision breeding for cassava.

**Supplementary Information:**

The online version contains supplementary material available at 10.1186/s13007-023-01055-5.

## Background

In recent decades, advances in plant virology and next-generation sequencing technologies have facilitated the identification of novel viruses and the assembly of complete viral genomes [1, 2]. The increasing availability of full-length viral genome sequences has contributed to the development of full-length viral DNA or cDNA infectious clones, which are used to study the viral pathogenesis [3]. Furthermore, many plant viruses have been engineered as versatile viral vectors to deliver exogenous sequences into host plants for protein overexpression, gene silencing, and genome editing [4–6]. The use of plant viral vectors offers significant advantages, including rapid expression of desired products at the whole plant level in a short period of time through simple virus inoculation [4, 6]. Cauliflower mosaic virus was first used as a virus-mediated protein overexpression (VOX) vector more than three decades ago [7]. Since then, numerous DNA and RNA viruses have been modified as VOX vectors. These vectors are used not only for the production of important functional or pharmaceutical proteins, but also for the screening of insecticidal proteins and the identification of pathogenic factors of viruses, fungi and bacteria in host plants [4, 8–10]. Subsequently, plant viruses can be engineered as virus-induced gene silencing (VIGS) vectors, which can induce the transient down-regulation of endogenous plant genes based on the post-transcriptional gene silencing machinery [11]. The VIGS systems have been used to characterize plant genes involved in plant development, metabolic regulation, and abiotic and biotic stresses in plants, especially in species that are recalcitrant to genetic transformation [12]. In just a decade, clustered regularly interspaced short palindromic repeats (CRISPR)/CRISPR associated (Cas)-mediated genome editing has revolutionized genetic engineering by enabling precise genome modification in cells or organisms [13]. Recently, the delivery of CRISPR-Cas components has demonstrated the utility of plant viral vectors for gene editing in plant host species, also known as virus-induced genome editing (VIGE) [14, 15]. There are two main strategies for the delivery of CRISPR/Cas components using plant viral vectors. One involves the expression of one or more single guide RNAs (sgRNAs) using a plant viral vector in Cas nuclease-transgenic plants [16–18]. Alternatively, the sgRNA and Cas nuclease can be delivered to the whole plant using a single viral vector or two synergistic viral vectors [19–21]. A major challenge of this method is that many plant viruses cannot accommodate the large size of the Cas9 gene due to their limited cargo capacity. VIGE is currently used not only as a transient tool to evaluate the specificity and efficiency of sgRNA designs, but also as a rapid platform to generate transgenic or transgene-free gene-edited plants by bypassing genetic transformation [15]. To date, the number of available VIGE vectors is less than that of VOX and VIGS vectors in plants [11, 15]. VIGE is expected to overcome the limitations of gene delivery for genome editing in various crop species, and make significant contributions to plant functional genome studies and crop breeding.

Cassava (*Manihot esculenta* Crantz; Euphorbiaceae) is not only a staple food crop in tropical regions but also an important source of livestock feed, industrial starch, and ethanol in many tropical Asian and African countries [22–24]. Genetic transformation protocols of cassava have been used to study gene function and generate transgenic germplasm with desirable agronomic traits related to yield, quality, and resistance to various stresses [25, 26]. However, the tissue culture and regeneration procedures involved in cassava genetic transformation are still laborious and time-consuming. The sequencing of the cassava genome and the increasing availability of genetic data have highlighted the need for faster and more efficient forward or reverse genetic tools for functional genomics research and genetic improvement of cassava [27, 28]. To date, two mosaic geminiviruses, namely African cassava mosaic virus (ACMV) and East African cassava mosaic virus (EACMV), and a cassava common mosaic virus (CsCMV; *Potexvirus* in the family *Alphaflexiviridae*) have been developed as VIGS vectors for cassava gene functional studies [28–30]. Among them, the CsCMV-based VIGS vector we constructed has been widely used to study the function of cassava genes involved in pathogen and pest resistance, abiotic stress tolerance, and cyanogenic glycoside biosynthesis [31, 32]. This vector offers several advantages such as mild infection symptoms, high silencing efficiency and rapid Nimble Cloning (NC) of target fragments into the viral genome [28, 33]. However, there are no VOX and VIGE vectors available in cassava.

**Fig. 1:**
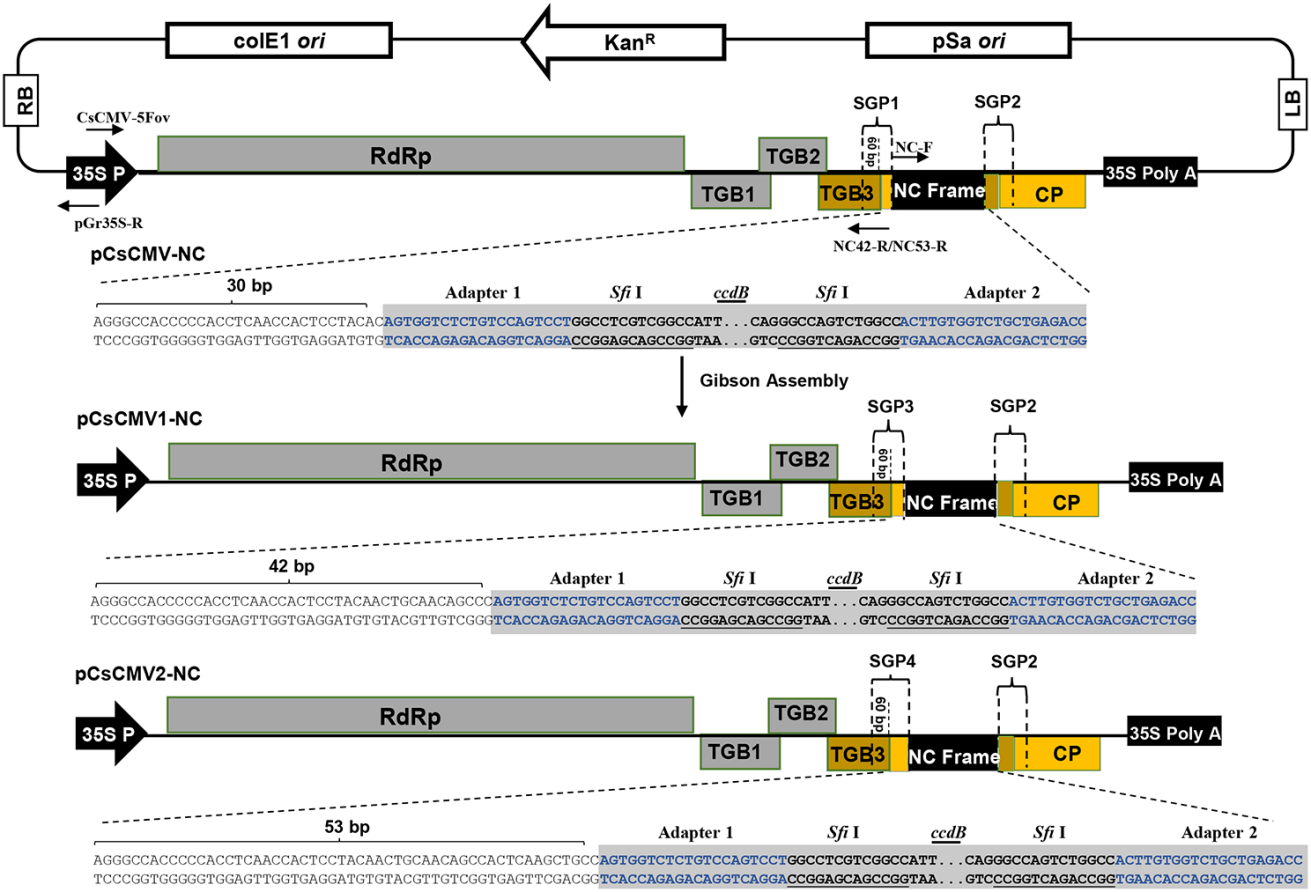
Schematic representation of two modified CsCMV-NC expression vectors The previous pCsCMV-NC plasmid contains a duplicated 90-bp putative CsCMV-CM *CP* subgenomic promoter (SGP1) (GenBank accession number MW175326; nt 5,534–5,623) and a Nimble Cloning (NC) frame sequence (adapter 1–*Sfi *I–*ccdB*–*Sfi *I–adapter 2). The SGP1 consisted of a 60-bp region upstream and a 30-bp region downstream of the *CP* start codon, respectively. In addition, SGP2 represented an authentic CP promoter. The two PCR products obtained from amplification of the pCsCMV-NC template using the CsCMV-5Fov/NC42-R or NC53-R primers, together with the NC-F/pGr35S-R primers, were assembled to generate the pCsCMV1-NC and pCsCMV2-NC constructs, respectively. The length of the sequence downstream of the *CP* start codon in SGP1 within pCsCMV-NC was extended from 30 to 42 and 53 bp. The resulting SGPs were denoted as SGP3 in pCsCMV1-NC and SGP4 in pCsCMV2-NC

CsCMV is a typical member of the genus *Potexvirus*, characterized by its single-stranded, positive-sense genomic RNA. Potato virus X (PVX) and foxtail mosaic virus (FoMV) are notable potexviruses that have been extensively used as viral vectors for gene overexpression, gene silencing, and genome editing in dicots and monocots [18, 34–39]. In this study, we first used the VIGS vector pCsCMV-NC for green fluorescent protein (GFP) expression in cassava plants, but the expression level was low. To increase the expression level of the target protein in cassava plants, we modified the pCsCMV-NC vector into the VOX vector named pCsCMV2-NC by extending the length of the duplicated putative subgenomic promoter (SGP1) of the CsCMV *CP* gene (Fig. 1). Subsequently, we used the pCsCMV2-NC vector to overexpress a bacterial phytoene synthase gene (*crtB*) [40] and a gene encoding the type III effector XopAO1 from *Xam* [41] for viral infection tracking, carotenoid biofortification, and virulence factor identification in cassava plants, respectively. In addition, we used pCsCMV2-NC to separately deliver two single guide RNAs (sgRNAs) targeting the cassava phytoene desaturase gene (*MePDS*) into Cas9-overexpressing (Cas9-OE) transgenic cassava lines. This resulted in distinct photobleaching leaf phenotypes caused by CRISPR/Cas9-mediated deletion mutations in the *MePDS* locus.

## Results

### Modification of pCsCMV-NC as a protein overexpression vector in cassava plants

**Fig. 2:**
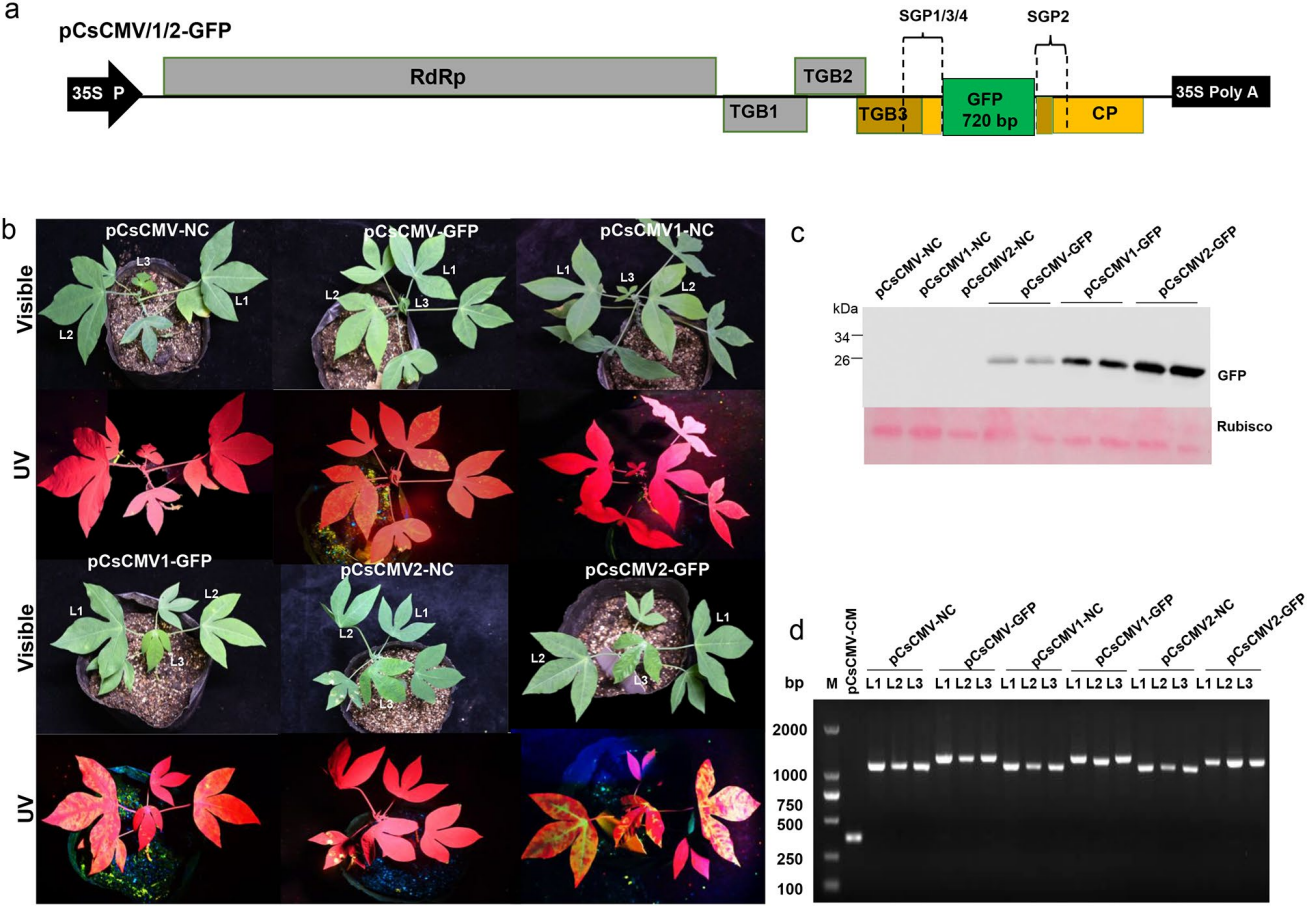
**Comparison of GFP expression and fluorescence levels in cassava plants infected with CsCMV-GFP, CsCMV1-GFP, or CsCMV2-GFP** (**a**) Schematic representation of pCsCMV-GFP, pCsCMV1-GFP, and pCsCMV2-GFP expression vectors. (**b**) GFP fluorescence in cassava plants infected with CsCMV-GFP, CsCMV1-GFP, or CsCMV2-GFP under ultraviolet light at 20 dpi. (**c**) Western blot analysis of GFP abundance in cassava plants infected with CsCMV-GFP, CsCMV1-GFP, or CsCMV2-GFP using an anti-GFP antibody at 20 dpi. Ponceau S staining of the large subunit of Rubisco was used as a loading control. (**d**) RT-PCR analysis of the stability of the inserted *GFP* gene in CsCMV-GFP, CsCMV1-GFP, or CsCMV2-GFP (L1 to L3: first, second, and third leaves above the inoculated leaves). The empty vectors pCsCMV-NC, pCsCMV1-NC, pCsCMV2-NC, and pCsCMV-CM were used as the controls

To evaluate the potential of the previous pCsCMV-NC VIGS vector for protein expression in cassava, we cloned the GFP coding sequence downstream of SGP1 in pCsCMV-NC using NC [33], resulting in a pCsCMV-GFP vector (Fig. 2a). At 20 days post-inoculation (dpi), weak GFP fluorescence was detected in the systemic leaves infected with CsCMV-GFP when examined under UV light (Fig. 2b). However, the fluorescence signal intensity did not increase with the duration of the infection period. The weak fluorescence indicated relatively low GFP expression in CsCMV-GFP- infected cassava leaves (Fig. 2c). In the potexvirus-based expression vector, increasing the length of the duplicated SGP enhanced the expression of foreign proteins [34]. In pCsCMV-NC, the duplicated SGP1 sequence consisted of 60 bp upstream and 30 bp downstream of the CsCMV *CP* start codon. Therefore, we designed duplicated SGP3 and SGP4 with longer downstream regions (42 and 53 bp) of the *CP* start codon to generate two modified vectors, pCsCMV1-NC and pCsCMV2-NC (Fig. 1). The GFP-encoding gene was then inserted into the NC frame of each modified CsCMV expression vector, resulting in pCsCMV1-GFP and pCsCMV2-GFP vectors (Fig. 2a). At 20 dpi, UV illumination revealed that green fluorescence was stronger in systemic leaves of CsCMV2-GFP-infected plants compared to those infected with CsCMV1-GFP or CsCMV-GFP after agroinfiltration (Fig. 2b). To assess GFP protein expression in the first, second, and third systemic leaves (L1-L3), Western blot analysis was performed using an anti-GFP antibody. Consistent with the observed intensity of green fluorescence, CsCMV2-GFP mediated higher GFP protein expression in the systemic leaves than CsCMV1-GFP or CsCMV-GFP (Fig. 2c). This indicates that extending the length of the duplicated SGP1 enhances the expression of foreign proteins. To evaluate the stability of the inserted *GFP* in CsCMV-GFP, CsCMV1-GFP, and CsCMV2-GFP, we performed RT-PCR analysis on L1, L2, and L3 leaves where green fluorescence was detected at 20 dpi. Primers CsCMV5416F/5730R, which annealed to sequences flanking the NC frame, were used. No additional PCR products were observed in any of the leaves, except for the expected bands, confirming the retention of the inserted GFP sequence (Fig. 2d). Thus, increasing the length of the duplicated SGP1 did not affect the stability of the pCsCMV2-NC vector during virus infection. The pCsCMV2-NC vector has the potential to overexpress genes of interest in cassava.

### CsCMV-mediated overexpression of the bacterial phytoene synthase gene (*crtB*) for the carotenoid biofortification in cassava plants

**Fig. 3:**
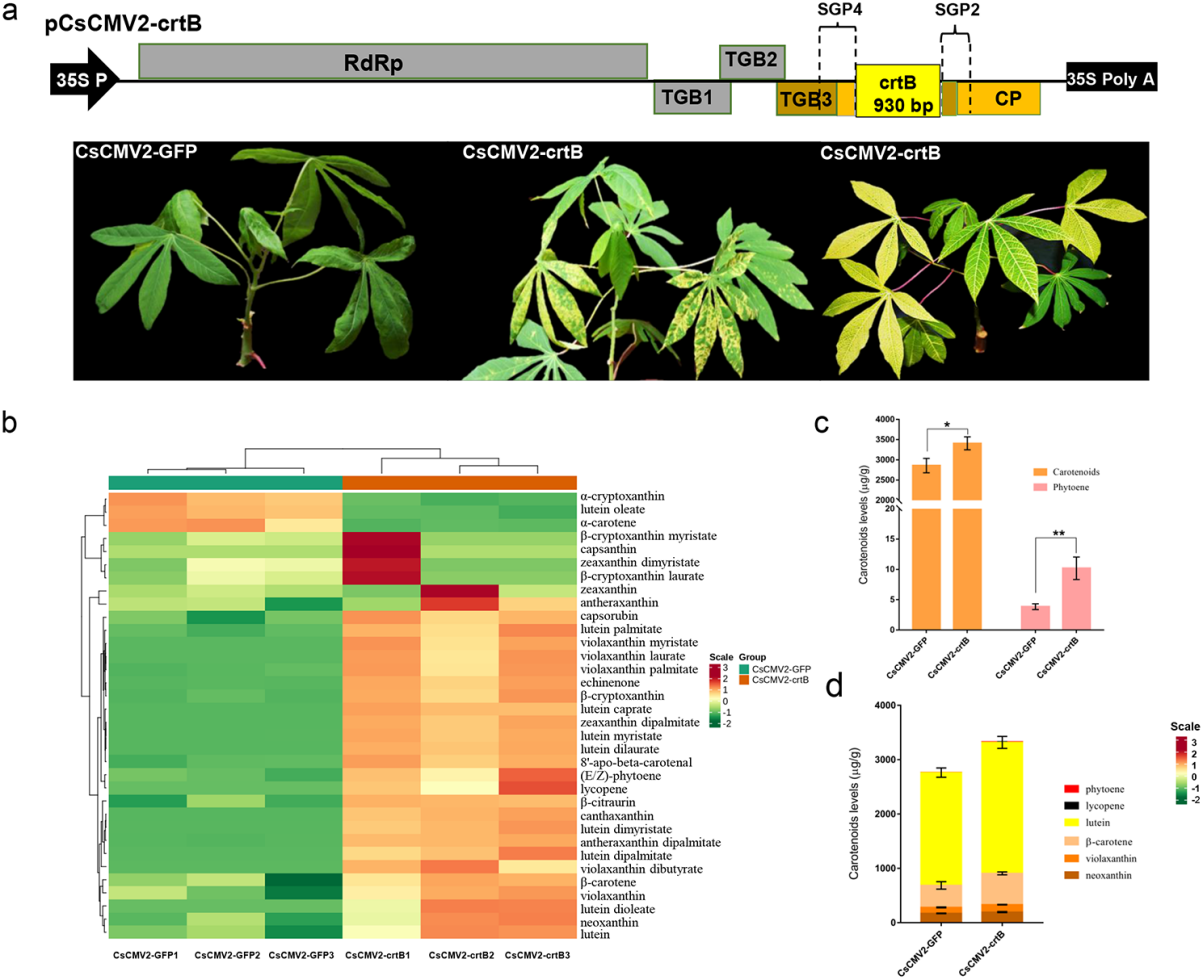
**Carotenoid accumulation in cassava leaves infected with CsCMV2-crtB** (**a**) Schematic representation of the pCsCMV2-crtB expression vector. (**b**) Phenotypes of cassava leaves agroinfiltrated with CsCMV2-crtB or CsCMV2-GFP at 20 dpi. (**c**) Heat map of the differentially accumulated carotenoids in yellow cassava leaves from CsCMV2-crtB-infected cassava plants and symptomatic CsCMV2-GFP-infected leaves. (**d**) Comparative analysis of carotenoid content in the systemic leaves of cassava plants infected with CsCMV2-crtB and CsCMV2-GFP at 20 dpi. Error bars indicate the standard deviation of three independent samples. Significant differences were determined by Student’s *t*-test (^*^P < 0.05 and ^**^P < 0.01)

To enhance carotenoid biofortification in cassava plants, we used the pCsCMV2-NC vector to overexpress the *crtB* gene in cassava. The crtB coding sequence was cloned into the pCsCMV2-NC vector using the NC system, resulting in pCsCMV2-crtB (Fig. 3a). As expected, the systemic leaves of cassava plants agroinfiltrated with CsCMV2-crtB showed yellow mosaic pattern or widespread areas of yellowing, indicating altered pigmentation (Fig. 3b). Metabolomics analysis revealed the differential accumulation of 34 carotenoids between the yellowing cassava leaves infected with CsCMV2-crtB and the control leaves infected with CsCMV2-GFP (Fig. 3c and Additional file 2: Table S1). Among them, 22 carotenoids showed significantly higher abundance in the CsCMV2-crtB-infected yellowing leaves compared to the CsCMV2-GFP-infected control leaves (Additional file 2: Table S2) Notably, CsCMV2-mediated crtB overexpression resulted in phytoene overaccumulation (Fig. 3c), which led to a significant increase in the levels of downstream endogenous carotenoids, including lycopene, β-carotene, lutein, violaxanthin, and zeaxanthin (Fig. 3d).

### Investigation of a virulence effector of the cassava bacterial blight pathogen using the pCsCMV2-NC vector

**Fig. 4:**
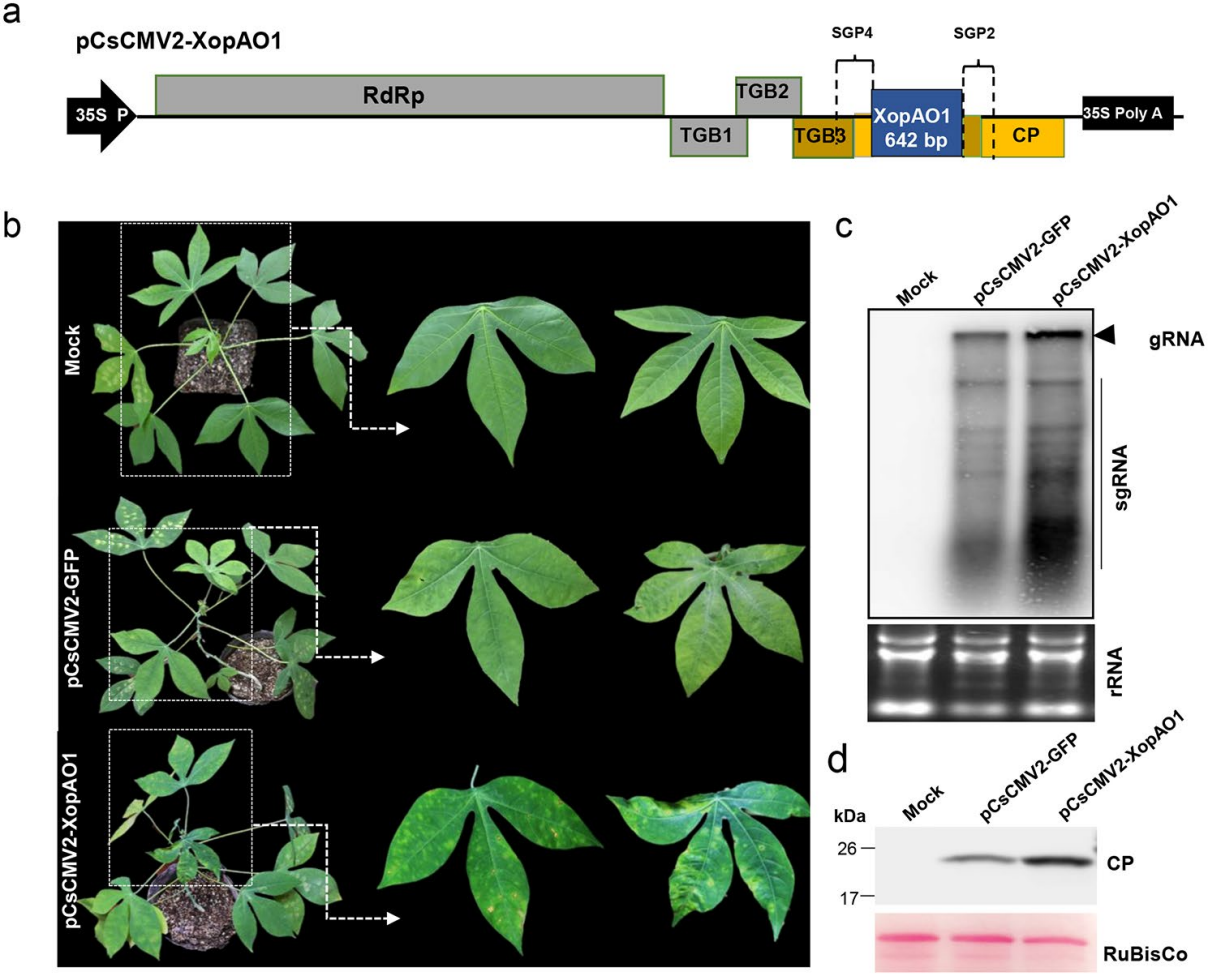
**Overexpression of the** ***Xanthomonas axonopodis*** **pv.** ***manihotis XopAO1*** **gene in cassava using the pCsCMV2-NC vector** (**a**) Schematic representation of the pCsCMV2-XopAO1 expression vector. (**b**) Systemic symptoms on cassava plantlets (cultivar SC8) agroinfiltrated with CsCMV2-XopAO1 or CsCMV2-GFP at 20 dpi. (**c**) Northern blot analysis of the accumulation of viral genomic and subgenomic RNAs in the systemic leaves of plants infected with CsCMV2-XopAO1 or CsCMV2-GFP at 20 dpi using a CsCMV *CP*-specific RNA probe. Ethidium bromide-stained rRNA is shown in the lower panels. (s) Western blot analysis of CsCMV CP expression in cassava plants infected with CsCMV2-XopAO1 or CsCMV2-GFP using an anti-CP antibody. The Ponceau S staining of the large subunit of Rubisco was used as a loading control

To investigate the role of the pCsCMV2-NC vector in study of virulence effectors of cassava pathogens, we cloned the full-length coding sequence of an important type III effector, *XopAO1*, from *Xam*, into the pCsCMV2-NC vector (Fig. 4a). Cassava leaves were agroinfiltrated with the resulting construct, pCsCMV2-XopAO1. At 20 dpi, plants inoculated with CsCMV2-XopAO1 exhibited severe mosaic, distorted, and necrotic symptoms on systemic leaves, in contrast to the relatively mild mosaic symptoms observed in control plants agroinfiltrated with CsCMV2-GFP (Fig. 4b). Northern and Western blot analyses revealed significantly higher expression levels of CsCMV genomic and subgenomic RNAs, and CP protein in CsCMV2-XopAO1-infected cassava plants compared to CsCMV2-GFP-infected controls (Fig. 4c, d). These results suggest that XopAO1 serves as a critical virulence effector that exacerbates disease symptoms in cassava.

### CsCMV2-induced genome editing in cassava

**Fig. 5:**
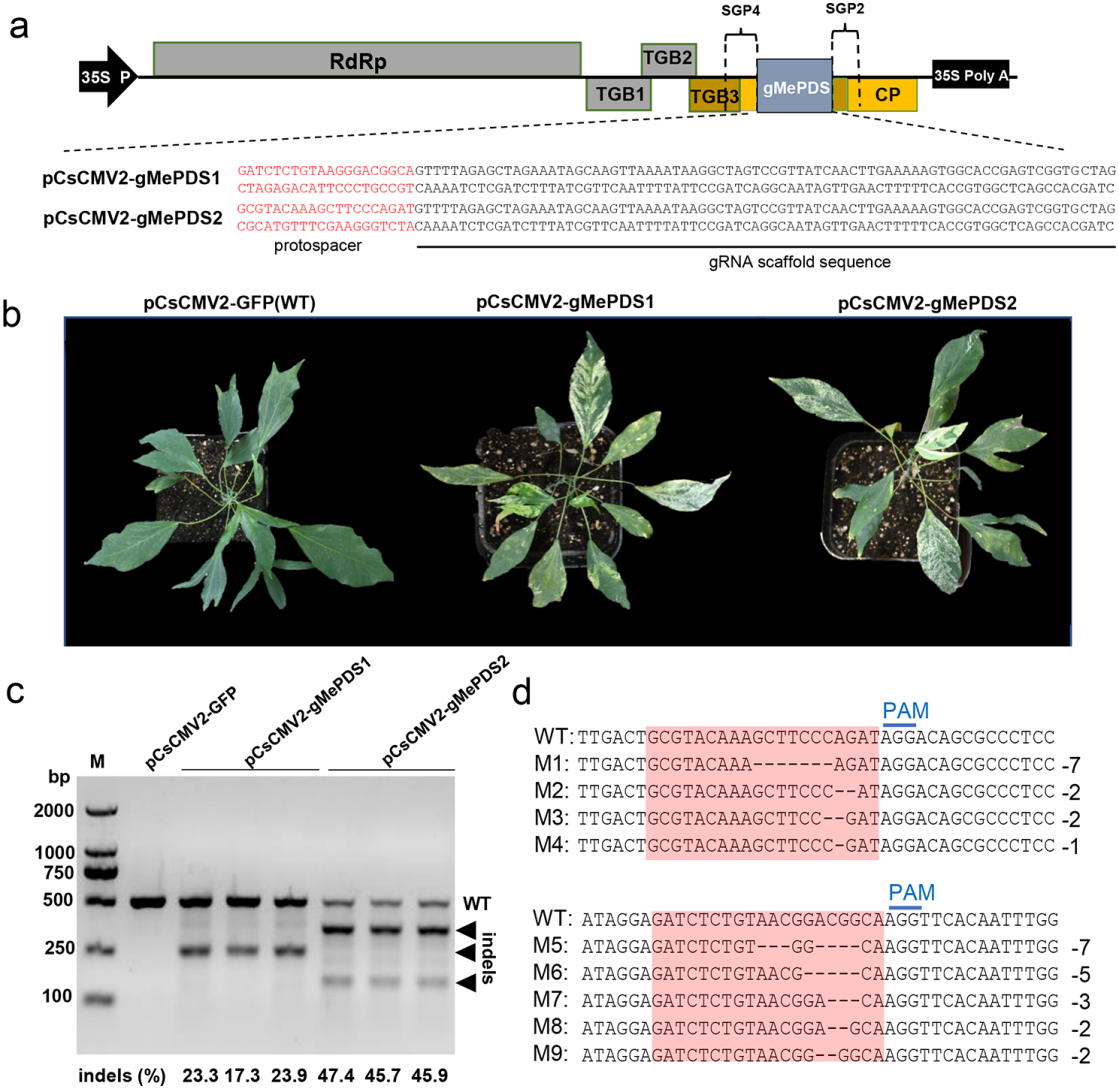
**CsCMV-mediated genome editing using the CRISPR/Cas9 system in cassava.** (**a**) Schematic representation of the pCsCMV2-gMePDS1 and -gMePDS2 vectors. The protospacer region sequences (marked in red) and the gRNA scaffold sequences (underlined) were cloned downstream of SGP4 into the pCsCMV2-NC vector. (**b**) Representative photobleaching phenotypes in Cas9-overexpressing (Cas9-OE) transgenic cassava lines agroinfiltrated with CsCMV2-gMePDS1 or -gMePDS2 at 35 dpi. The CsCMV2-GFP-infected plants were used as the controls. (**c**) The T7 endonuclease 1 (T7E1) mismatch detection assay of CsCMV2-gMePDS1 or -gMePDS2-induced genome editing. Targeted *MePDS* gene mutagenesis was detected in the photobleached leaves of three representative Cas9-OE transgenic cassava lines agroinfiltrated with CsCMV2-gMePDS1 or -gMePDS2. Arrows indicate the T7EI cleavage products. The indel rate (%) was calculated using the Image J software. (**d**) Sanger sequencing of wild-type (WT) and mutant versions (M1-M9) of the *MePDS* gene from photobleached cassava leaf area via CsCMV2-gMePDS1 or -gMePDS2-induced genome editing. The target/mutated sequences are highlighted in light red. The protospacer-associated motif (PAM) is underlined in blue. The number of different nucleotide deletions in the *MePDS* target sites is indicated on the right

To evaluate the feasibility of using pCsCMV2-NC to deliver specific sgRNAs to Cas9-OE transgenic cassava line, we inserted two previously reported synthetic gRNA scaffolds (gMePDSs) targeting two sites within MePDS exon 13 into the pCsCMV2-NC vector under the control of SGP4 [42], resulting in pCsCMV2-gMePDS1 and pCsCMV2-gMePDS2 (Fig. 5a). The upper uninoculated leaves of Cas9-OE transgenic cassava lines, agroinfiltrated with CsCMV2-gMePDS1 or -gMePDS2, initially exhibited mild photobleaching at 20 dpi, but subsequently developed a severe albino phenotype at 35 dpi (Fig. 5b). To confirm on-target genome editing and estimate the editing efficiency, a T7 endonuclease I (T7EI) mismatch cleavage assay was performed. First, a 504-bp fragment flanking the target genomic *MePDS* sequence was amplified by PCR using genomic DNA extracted from photobleached leaves of Cas9-OE plants infected with CsCMV2-gMePDS1 or -gMePDS2, and the PCR products were digested with T7EI. The T7EI assays revealed partial cleavage of the 504-bp MePDS PCR products from Cas9-OE transgenic cassava plants infected with CsCMV2-gMePDS1 and -gMePDS2, resulting in one or two smaller DNA bands. Only a single smaller T7EI-digested band was detected in CsCMV2-gMePDS2-infected Cas9-OE cassava plants because the editing site was located in the middle of the 504-bp *MePDS* fragment (Fig. 5b). No T7EI-digested DNA fragments were observed in the CsCMV2-GFP-infected control plants. The presence of the T7EI cleaved band indicated that the delivery of sgRNAs via CsCMV2-gMePDS1 or -gMePDS2 infection resulted in insertions and deletions (indels) in the *MePDS* gene. Quantitative analysis of the T7EI-digested products from three independent plants revealed that the efficiency of CsCMV2-gMePDS1 and -gMePDS2-induced gene editing (indel percentage) ranged from 17.3 to 23.9% and 45.7–47.4%, respectively. Sanger sequencing of the *MePDS* target sequence confirmed the deletion of 1 to 7 bp in the region upstream of the protospacer adjacent motif (PAM) sites (Fig. 5c and Additional file 1: Fig. S1). Thus, the pCsCMV2-NC vector proves to be an efficient tool for sgRNA delivery in CRISPR/Cas9-based genome editing of cassava.

## Discussion

We previously developed a CsCMV-NC VIGS vector by incorporating the duplicated 90-bp putative SGP of the CsCMV CP gene and the NC cloning frame into the viral genome, which allowed efficient silencing of endogenous genes in cassava [28]. However, when this vector was used to express the GFP-encoding gene, only weak GFP fluorescence and expression were observed in the systemic leaves of cassava plants infected with CsCMV-GFP. Various strategies have been used to enhance the expression of heterologous proteins in plants using potexvirus-based vectors, including optimizing the length of the SGP, replacing the SGP with sequences from related poxviruses, incorporating gene expression enhancer elements, and mutating viral genes to promote virus accumulation [34, 39, 43–45]. Previous studies using FoMV-mediated VOX vectors have shown that increasing the length of the duplicated SGP2 sequence can enhance the expression of heterologous proteins [34]. In this study, we modified the CsCMV-NC vector by extending the downstream region of the *CP* start codon from 30 to 42 and 53 bp, resulting in the generation of two modified vectors, pCsCMV1-NC and pCsCMV2-NC. Analysis of protein expression using these vectors revealed that the extension of the sequence downstream of the *CP* start codon by 53 nt positively influenced GFP expression in cassava leaves infected with CsCMV2-GFP. We also investigated whether the longer duplicated SGP promoters resulted in loss of foreign genes during infections due to homologous recombination between duplicated sequences [44, 45]. Our RT-PCR assays confirmed that the inserted *GFP* sequence was retained and not lost or deleted in all systemic cassava leaves showing green fluorescence signals upon CsCMV-GFP, CsCMV1-GFP, or CsCMV2-GFP infections. Therefore, the improved CsCMV2-NC-based vector is well suited to achieve systemic expression of heterologous proteins in cassava plants. In addition. CsCMV2-GFP can be used to monitor viral infections and movement in CsCMV-host interactions and to evaluate CsCMV resistance in cassava breeding programs.

The *Pantoea ananatis crtB* gene encodes a phytoene synthase that catalyzes the condensation of two geranylgeranyl diphosphate (GGPP) molecules, thereby initiating the carotenoid biosynthesis pathway with the formation of phytoene [40]. Previous studies have shown that tobacco etch virus, tobacco mosaic virus, PVX, and zucchini yellow mosaic virus-mediated expression of the *crtB* gene in *Arabidopsis thaliana*, tobacco, tomato, lettuce leaves, and zucchini fruits resulted in the accumulation of phytoene. This accumulation triggered the differentiation of chloroplasts into chromoplasts and subsequently promoted the biosynthesis and accumulation of carotenoids [46–49]. Virus-mediated enhancement of carotenoid biosynthesis offers a promising biotechnological approach to improve the nutritional value of green food and forage crops. Indeed, cassava leaves are widely consumed as a traditional food and livestock feed in Central Africa, South and Southeast Asia, and Brazil due to their high protein, vitamin, and mineral content [50]. In this study, we used the CsCMV2-NC vector to facilitate crtB expression in cassava leaves and manipulate carotenoid biosynthesis. Expression of crtB significantly increased the levels of 22 carotenoid compounds, including phytoene and downstream metabolites such as beta-carotene, in cassava leaves compared to control leaves. These carotenoid-enriched cassava leaves are a potential source for meeting the body’s vitamin A requirements, especially in certain underdeveloped cassava-growing countries.

Cassava bacterial blight, caused by *Xam*, is a widespread bacterial disease that poses a significant threat to cassava growth and production [51]. Identification of the pathogenic effectors of *Xam* is crucial to understand the molecular mechanisms underlying disease development and to develop effective strategies to control cassava bacterial blight [41].Current methods for screening *Xam* virulence effectors primarily involve knockout mutations in effector genes. However, it is generally observed that mutations in effector genes do not significantly alter bacterial virulence, possibly due to functional redundancy among protein effectors [52]. To rapidly identify virulence effectors, potexvirus-based vectors have been used for systemic expression of effector gene candidates from various phytopathogens in plants [10]. In our study, CsCMV-mediated expression of XopAO1 resulted in more severe viral disease symptoms and increased accumulation of CsCMV compared to the control CsCMV2-GFP. Previous research has identified XopAO1 as an important virulence effector that suppresses both pathogen-associated molecular pattern-triggered immunity and effector-triggered immunity in cassava during *Xam* infection [41].These results suggest that the enhanced viral disease symptoms and substantial virus accumulation in cassava plants infected with CsCMV2-XopAO1 may be due to overexpression of XopAO1, which suppresses plant immune responses. Therefore, our modified pCsCMV2-NC vector has significant potential for the identification and characterization of virulence effectors of *Xam* and other cassava pathogens.

The CRISPR/Cas9-based gene editing system was initially established in cassava by expressing gMePDS1 or gMePDS2 targeting two sites within *MePDS* exon 13 using *Agrobacterium*-mediated transformation [42]. Recent studies have demonstrated successful CRISPR/Cas9-mediated mutagenesis of starch or cyanogen glycoside biosynthesis-related genes in transgenic cassava plants, resulting in reduced starch content and elimination of toxic cyanogen glycosides [53–55]. Compared with the *Agrobacterium tumefaciens*-based transformation method, plant virus-mediated sgRNA delivery systems offer a time-saving and less labor-intensive approach, avoiding the need for tissue culture and regeneration. In this study, we delivered gMePDS1 or gMePDS2 into Cas9-OE transgenic cassava plants using the pCsCMV2-NC vector. Infection of cassava plants with CsCMV2-gMePDS1 or CsCMV2-gMePDS2 resulted in the observation of albino phenotypes. The T7EI mismatch detection assay indicated a higher genome editing efficiency for CsCMV2-gMePDS1 compared to CsCMV2-gMePDS2. Sanger sequencing of the *MePDS* target sequence confirmed the presence of deletion mutations upstream of the PAM site. These results suggest that our approach offers a promising alternative to genetic transformation-based methods for genome editing in cassava.

For non-seed-transmitted viruses such as CsCMV, there are currently two main strategies for acquiring heritable mutations by VIGE. The first strategy involves the in vitro regeneration of virus-infected plant tissues with edited genes, which has been successfully employed in PVX, barley stripe mosaic virus, and sonchus yellow net rhabdovirus (SYNV)-mediated gene editing experiments [18, 20, 56]. Existing cassava regeneration protocols can be used to obtain edited progeny from CsCMV-infected cassava tissues using VIGE constructs through tissue culture [25]. The second method involves the production of gene-edited seeds by delivering gRNA fused to the mobile Flowering Locus T (*FT*) mRNA sequence into germline cells. Studies have shown that FT-fused gRNAs delivered by PVX, tobacco rattle virus, and cotton leaf crumple virus induced heritable genome editing in *A. thaliana*, *Nicotiana benthamiana*, and *Nicotiana attenuate* when the gRNAs [16, 18, 57]. In addition, the SYNV and tomato spotted wilt virus-based negative strand RNA viral vectors have been used to deliver all CRISPR-Cas9 components into plant cells to generate the heritable transgene-free edited crop species [19, 20]. However, expression of the entire CRISPR-Cas9 construct in systemic tissues using potexvirus-based vectors is challenging due to the limited viral cargo capacity [58]. To overcome this limitation, smaller versions of Cas effector proteins, such as Cas12f, have recently been reported as potential alternatives to the large size of Cas9 in the future [59, 60].

## Conclusions

In summary, the modified CsCMV-based vector can be used not only for heterologous gene expression but also for genome editing in cassava. The broader applications of this vector will greatly facilitate functional genomics studies, biotechnology applications and genetic modification in cassava.

## Methods

### Plant materials and growth conditions

In this study, 3-week-old and 5-month-old cassava plants (cultivar SC8) were used for the analysis of CsCMV-mediated protein overexpression. In addition, Cas9-OE transgenic cassava lines were generated by transforming a *pYAO*:hSpCas9 binary vector into friable embryogenic calli of cassava cultivar SC8 by *Agrobacterium*-mediated genetic transformation in our previous study [61, 62]. About 5-week-old transgenic plantlets were subjected to agroinfiltration for CsCMV-induced genome editing experiments. All plants were grown in a greenhouse at 23°C under a photoperiod of 16 h of light followed by 8 h of dark.

#### Viral vector construction

The previously generated pCsCMV-NC plasmid [28] was used as a template for the PCR amplification using CsCMV-5Fov/NC42-R and NC-F/pGr35S-R primer pairs. The resulting PCR products, which contained overlapping regions, were assembled to generate pCsCMV1-NC following the instructions of the Gibson Assembly Cloning Kit (NEB). The SGP3 sequence in pCsCMV1-NC spanned 42 nucleotides (nts) downstream of the *CP* start codon. Similarly, two DNA fragments with overlapping regions were amplified via PCR using the pCsCMV-NC plasmid as the template and the CsCMV-5Fov/NC53-R and NC-F/pGr35S-R primer pairs, resulting in the construction of pCsCMV2-NC. The SGP4 sequence in pCsCMV2-NC included 53 nts downstream of the *CP* start codon. For the VOX plasmids, the GFP coding sequence (GenBank accession: MK896905) was PCR-amplified from the pPLDMV-GFP plasmid [63] and inserted into the pCsCMV-NC, pCsCMV1-NC, and pCsCMV2-NC vectors through NC to generate pCsCMV-GFP, pCsCMV1-GFP, and pCsCMV2-GFP, respectively. In addition, the *P. ananatis* carotenoid biosynthesis gene crtB (GenBank accession: D90087) was synthesized (Sangon Biotech) and subsequently inserted into pCsCMV2-NC to construct pCsCMV2-crtB. The sequence of XopAO1 (GenBank accession: CP083575.1) was PCR-amplified from *Xam* genomic DNA and cloned into pCsCMV2-NC to generate pCsCMV2-XopAO1. The NC procedure was performed as previously described [33, 64]. To target the cassava phytoene desaturase gene via VIGE, two previously reported 99-nt gRNA scaffolds (gMePDSs) were synthesized (Sangon Biotech) [42], and then amplified by PCR using primers containing the *Sfi* I restriction enzyme site. Each PCR product and the pCsCMV2-NC vector were digested with *Sfi* I, purified, and ligated. The resulting vectors were designated as the pCsCMV2-gMePDS1 and pCsCMV2-gMePDS2.

All primers used for viral vector construction are listed in Additional file 2: Tables S3 and S4. Prior to the transformation of *A. tumefaciens* GV3101 along with the pSoup helper plasmid, the accuracy of each CsCMV-based construct was confirmed by Sanger sequencing. The primer sequences used for constructing viral vectors are listed in Additional file 2: Table S3 and S4.

### Agroinfiltration of cassava plants

The *A. tumefaciens* suspensions were prepared and the infiltration of cassava plants performed according to the previously described protocol [28]. Briefly, *A. tumefaciens* strain GV3101 containing each construct was grown overnight at 28°C in LB supplemented with kanamycin (50 mg/L) and rifampicin (25 mg/L). The agrobacterial cells were then centrifuged at 2500×g for 5 min at 4°C and the pellet cells were resuspended in infiltration buffer (10 mM MgCl_2_, 10 mM 2-(N-morpholino) ethanesulfonic acid (pH 5.5) and 100 µM acetosyringone) to a final optical density at 600 nm (OD_600_) of 0.5. The bacterial suspension was kept at room temperature for 1–3 h in the dark prior to agroinfiltration. Infiltration was carried out at 8–10 points on either side of the main leaf vein using a 1 mL syringe.

### RT-PCR and Northern blot analyses

Total RNA was extracted from 100 mg cassava leaves using the RNAprep Pure Plant Kit (Tiangen Biotech). First-strand cDNA was synthesized from 1.0 µg total RNA using the HiScript III 1st Strand cDNA Synthesis Kit (Vazyme) with random hexamers and oligo(dT)_20_ VN primers. The presence of infection in each agroinoculated plant was confirmed by RT-PCR using primers CsCMV5416F/5730R, designed to anneal to regions flanking the NC frame as previously described [28]. To compare the accumulation of viral genomic and subgenomic RNAs between plants infected with CsCMV2-GFP and those infected with CsCMV2-XopAO1, Northern blot analyses were performed using the DIG Northern Starter Kit (Roche). Briefly, 1 µg of total RNA from cassava leaves was separated on a 1.2% agarose gel containing formaldehyde and then transferred to an Amersham Hybond-N+ nylon membrane (Cytiva) for hybridization. CsCMV CP-specific probes, labeled with digoxigenin, were used for hybridization. The probes were synthesized by in vitro transcription using a 690-bp PCR-amplified product of the CsCMV *CP* gene with the T7 promoter. The signals from the hybridization band were detected using the CDP-Star reagent in the kit and visualized using the ImageQuant LAS 4000 mini biomolecular imager (GE Healthcare).

### Western blot

Proteins were extracted from 0.1 g of cassava leaves using the method described by Wang et al. [65]. The presence of GFP and CsCMV CP proteins was detected by a western blotting assay. The assay involved the use of anti-GFP/CP monoclonal antibodies (Sangon Biotech) and horseradish peroxidase (HRP)-conjugated goat anti-rabbit IgG antibodies (Sangon Biotech), followed by visualization with a chemiluminescent HRP substrate (Millipore, USA).

### Fluorescence detection

The GFP fluorescence signal was detected in systemically infected cassava leaves using an ultraviolet lamp (LUYOR-3415RG; LUYOR Corporation).

### Carotenoid metabolite analysis

Tissue samples were prepared and carotenoid metabolites were extracted following a previously described method [66]. Carotenoid contents were quantified using the AB Sciex QTRAP 6500 LC-MS/MS platform from MetWare (http://www.metware.cn/). Each assay was performed in triplicate. Heatmaps with dendrograms were generated to visualize the results of the hierarchical cluster analysis (HCA) of the samples and metabolites. The HCA was performed using the R package pheatmap. Significant differences in carotenoid components between groups were determined based on fold change (FC) ≥ 2 or ≤ 0.5.

### T7 endonuclease I (T7EI) mismatch cleavage assay and Sanger sequencing

Cassava genomic DNA was extracted using the Plant Genomic DNA Kit (Tiangen, China). The T7EI assay performed according to the previously described protocols [14, 67]. Briefly, a 504-bp fragment of MePDS (Manes.05G193700.1) containing the target sites was amplified from genomic DNA (gDNA) using PrimeSTAR Max DNA Polymerase (Takara) and the MePDSF2/R2 primer pair [42]. The PCR products were purified from agarose gels using the FastPure Gel DNA Extraction Mini Kit (Vazyme). The purified PCR products (200 ng) were then denatured and reannealed in NEBuffer 2 (NEB) to generate heteroduplex DNA under the following reaction conditions: 95°C for 10 min, 85°C for 2 min, 75–25°C (with 10°C increments over 3 min), and 4°C for 10 min. The heteroduplex DNA was then incubated with 10 U of T7E1 enzyme (NEB) at 37°C for 1 h. The products of the T7EI reaction were analyzed by 2% agarose gel electrophoresis. Mutation rates were determined using Image J software (http://imagej.nih.gov/ij/) following previously described methods [68]. In addition, the purified PCR products were cloned into the pCE2 TA/Blunt-Zero vector using the TOPO cloning kit (Vazyme), and the mutations in the positive clones were identified through Sanger sequencing.

### Electronic supplementary material

Below is the link to the electronic supplementary material.

**Additional file 1: Figure S1.** Sanger sequencing chromatograms of the indels of MePDS target from leaves infected with CsCMV2-gMePDS1 or -gMePDS2. The arrowhead indicates the location of the indel. Different deletions are indicated by numbers.

**Additional file 2: Table S1.** Metabolomics data of carotenoids in cassava leaves infected with CsCMV2-crtB or CsCMV2-GFP

**Additional file 3: Table S2.** Differentially accumulated carotenoids in cassava leaves infected with CsCMV2-crtB vs. CsCMV2-GFP

**Additional file 4: Table S3.** Primers used for construction of the pCsCMV/1/2-NC vector

**Additional file 5: Table S4.** Primers used for cloning gene fragments into the pCsCMV/1/2-NC vector

## Data Availability

All data and materials are available upon request to W.S. (shenwentao@itbb.org.cn)

## Declarations

**Ethics approval and consent to participate**.

**Consent for publication**.
