# Supplementary figures and images for: Development of cassava common mosaic virus-based vector for protein expression and gene editing in cassava
Source: Plant Methods. 2023 Aug 3;19:78. doi: 10.1186/s13007-023-01055-5 (PMC10399001; doi:10.1186/s13007-023-01055-5)

## CsCMV2-gMePDS1

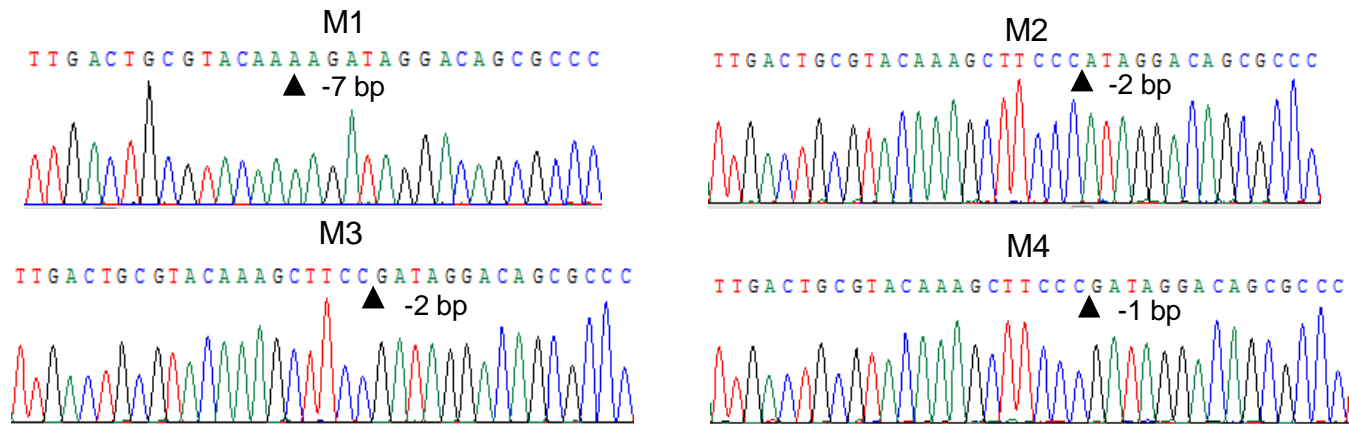

## CsCMV2-gMePDS2

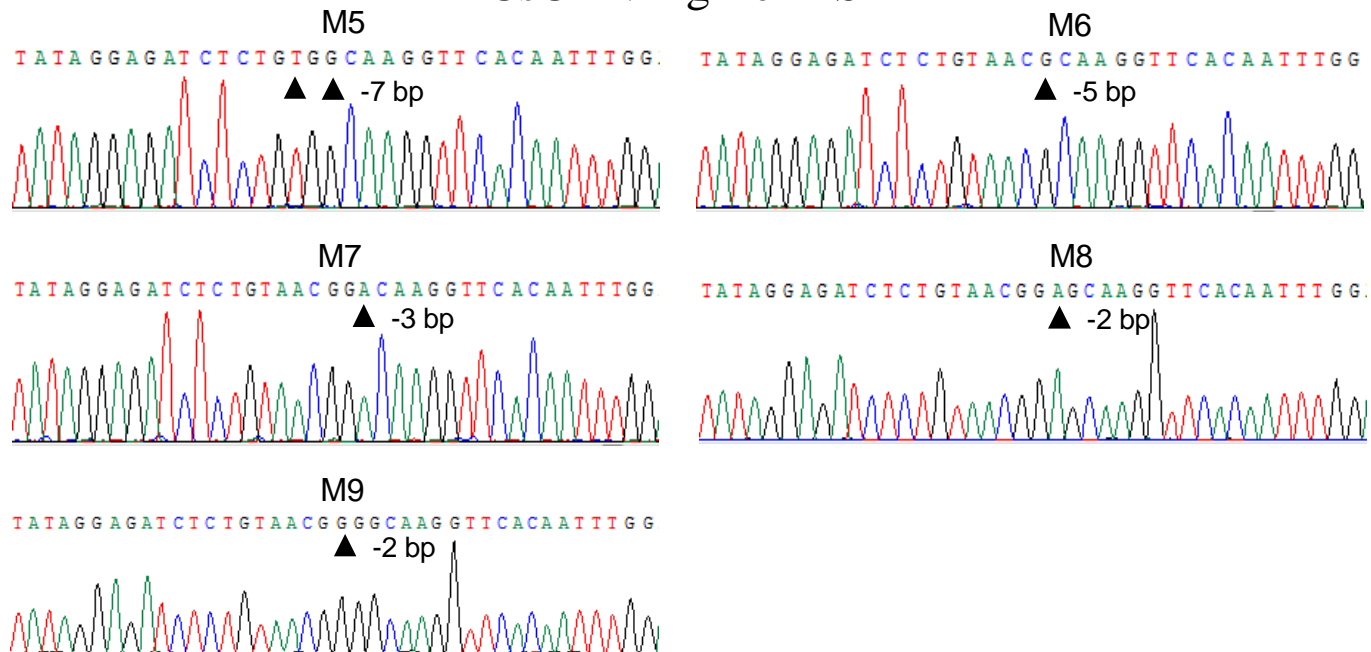

Supplement: Supplementary file 1 — Additional file 1: Figure S1. Sanger sequencing chromatograms of the indels of MePDS target from leaves infected with CsCMV2-gMePDS1 or -gMePDS2. The arrowhead indicates the location of the indel. Different deletions are indicated by numbers. [file 13007_2023_1055_MOESM1_ESM.pdf]
