# Supplementary material for: Development of cassava common mosaic virus-based vector for protein expression and gene editing in cassava
Source: Plant Methods. 2023 Aug 3;19:78. doi: 10.1186/s13007-023-01055-5 (PMC10399001; doi:10.1186/s13007-023-01055-5)
Supplement: Supplementary file 2 — Additional file 2: Table S1. Metabolomics data of carotenoids in cassava leaves infected with CsCMV2-crtB or CsCMV2-GFP [file 13007_2023_1055_MOESM2_ESM.pdf]

Additional file 1: Table S1- Metabolomics data of carotenoids in cassava leaves infected with CsCMV2-crtB or CsCMV2-GFP

Index	Compounds	Class	Q1 (Da)	Q3 (Da)	Molecular Weight	Ion mode	Ionization model	Formula	CsCMV2-GFP1	CsCMV2-GFP2	CsCMV2-GFP3	CsCMV2-crtB1	CsCMV2-crtB2	CsCMV2-crtB3
Carotenoid_01	α-carotene	carotenes	537.5	123.2	536.438232	positive	[M+H]+	C40H56	44.2858254	60.2183143	59.2194509	7.92587148	9.25852423	9.76136505
Carotenoid_07	ε-carotene	carotenes	537.6	123.2	536.438232	positive	[M+H]+	C40H56	N/A	N/A	N/A	N/A	N/A	N/A
Carotenoid_05	phytofluene	carotenes	543.5	81.2	542.4852	positive	[M+H]+	C40H62	N/A	N/A	N/A	N/A	N/A	N/A
Carotenoid_02	lycopene	carotenes	537.4	81	536.4382	positive	[M+H]+	C40H56	N/A	N/A	N/A	1.17029189	1.68477974	0.775609574
Carotenoid_03	γ-carotene	carotenes	537.4	177.3	536.4382	positive	[M+H]+	C40H56	N/A	N/A	N/A	N/A	N/A	N/A
Carotenoid_04	β-carotene	carotenes	537.6	177.1	536.4382	positive	[M+H]+	C40H56	326.801478	459.488033	427.78809	544.374213	585.480176	594.494569
Carotenoid_06	(E/Z)-phytoene	carotenes	545.3	81	544.5008	positive	[M+H]+	C40H64	3.34367101	3.95406868	4.27341454	10.0570139	12.1095374	8.39860314
Carotenoid_12	lutein palmitate	xanthophylls	789.8	533.5	806.8	positive	[M+H-18]+	C56H86O3	1.06769006	0.942971904	1.0832966	2.79836203	3.04022026	2.48833047
Carotenoid_10	lutein laurate	xanthophylls	733.5	533.3	750.5	positive	[M+H-18]+	C52H78O3	N/A	N/A	N/A	N/A	N/A	N/A
Carotenoid_13	lutein stearate	xanthophylls	817.8	533.5	834.8	positive	[M+H-18]+	C58H90O3	N/A	N/A	N/A	N/A	N/A	N/A
Carotenoid_14	5,6epoxy-luttein dilaurate	xanthophylls	749.6	549.5	948.6	positive	[M+H-200]+	C64H100O5	N/A	N/A	N/A	N/A	N/A	N/A
Carotenoid_16	5,6epoxy-lutein-caprate-palmitate	xanthophylls	805.4	549.4	976.4	positive	[M+H-172]+	C66H104O5	N/A	N/A	N/A	N/A	N/A	N/A
Carotenoid_19	lutein distearate	xanthophylls	817.9	533.5	1100.9	positive	[M+H-284]+	C76H124O4	N/A	N/A	N/A	N/A	N/A	N/A
Carotenoid_22	neochrome palmitate	xanthophylls	821.7	565.5	838.7	positive	[M+H-18]+	C56H86O5	N/A	N/A	N/A	N/A	N/A	N/A
Carotenoid_23	rubixanthin caprate	xanthophylls	707.7	535.6	706.7	positive	[M+H]+	C50H74O2	N/A	N/A	N/A	N/A	N/A	N/A
Carotenoid_24	rubixanthin laurate	xanthophylls	735.6	535.4	734.6	positive	[M+H]+	C52H78O2	N/A	N/A	N/A	N/A	N/A	N/A
Carotenoid_25	rubixanthin myristate	xanthophylls	763.5	533.5	762.5	positive	[M+H]+	C54H82O2	N/A	N/A	N/A	N/A	N/A	N/A
Carotenoid_26	rubixanthin palmitate	xanthophylls	791.7	535.4	790.7	positive	[M+H]+	C56H86O2	N/A	N/A	N/A	N/A	N/A	N/A
Carotenoid_31	violaxanthin palmitoleate	xanthophylls	837.7	745.6	836.7	positive	[M+H]+	C56H84O5	N/A	N/A	N/A	N/A	N/A	N/A
Carotenoid_32	violaxanthin dilaurate	xanthophylls	966.7	948.8	965.7	positive	[M+H]+	C64H101O6	N/A	N/A	N/A	N/A	N/A	N/A
Carotenoid_33	violaxanthin-myristate-caprate	xanthophylls	965.7	947.8	964.7	positive	[M+H]+	C64H100O6	N/A	N/A	N/A	N/A	N/A	N/A
Carotenoid_35	violaxanthin dimyristate	xanthophylls	1021.8	793.7	1020.8	positive	[M+H]+	C68H108O6	N/A	N/A	N/A	N/A	N/A	N/A
Carotenoid_36	violaxanthin-myristate-palmitate	xanthophylls	1050	793.8	1049	positive	[M+H]+	C70H112O6	N/A	N/A	N/A	N/A	N/A	N/A
Carotenoid_37	violaxanthin dipalmitate	xanthophylls	1077.9	821.7	1076.9	positive	[M+H]+	C72H116O6	N/A	N/A	N/A	N/A	N/A	N/A
Carotenoid_38	violaxanthin-myristate-oleate	xanthophylls	1075.9	847.7	1074.9	positive	[M+H]+	C72H114O6	N/A	N/A	N/A	N/A	N/A	N/A
Carotenoid_39	violaxanthin dioleate	xanthophylls	1129.9	829.8	1128.9	positive	[M+H]+	C76H120O6	N/A	N/A	N/A	N/A	N/A	N/A
Carotenoid_40	zeaxanthin myristoleate	xanthophylls	777.7	551.4	776.7	positive	[M+H]+	C54H80O3	N/A	N/A	N/A	N/A	N/A	N/A
Carotenoid_41	zeaxanthin palmitate	xanthophylls	807.8	551.5	806.8	positive	[M+H]+	C56H86O3	N/A	N/A	N/A	N/A	N/A	N/A
Carotenoid_42	zeaxanthin-caprate-laurate	xanthophylls	906.9	533.6	905.9	positive	[M+H]+	C60H121O4	N/A	N/A	N/A	N/A	N/A	N/A
Carotenoid_43	zeaxanthin dilaurate	xanthophylls	933.9	533.2	932.9	positive	[M+H]+	C64H100O4	N/A	N/A	N/A	N/A	N/A	N/A
Carotenoid_44	zeaxanthin-laurate-myristate	xanthophylls	962.7	733.5	961.7	positive	[M+H]+	C66H105O4	N/A	N/A	N/A	N/A	N/A	N/A
Carotenoid_47	zeaxanthin-myristate-palmitate	xanthophylls	1018.1	533.6	1017.1	positive	[M+H]+	C70H112O4	N/A	N/A	N/A	N/A	N/A	N/A
Carotenoid_49	zeaxanthin-palmitate-stearate	xanthophylls	1074.1	789.8	1073.1	positive	[M+H]+	C74H120O4	N/A	N/A	N/A	N/A	N/A	N/A
Carotenoid_50	zeaxanthin-oleate-palmitate	xanthophylls	1071.9	789.8	1070.9	positive	[M+H]+	C74H118O4	N/A	N/A	N/A	N/A	N/A	N/A
Carotenoid_34	violaxanthin-myristate-laurate	xanthophylls	993.8	975.7	992.8	positive	[M+H]+	C66H104O6	N/A	N/A	N/A	N/A	N/A	N/A
Carotenoid_11	lutein myristate	xanthophylls	761.8	533.5	778.8	positive	[M+H-18]+	C54H82O3	0.0313729341	0.0294942768	0.0300003867	1.61329483	1.59199119	1.40666617
Carotenoid_63	capsanthin	xanthophylls	585.5	109.1	584.871	positive	[M+H]+	C40H56O3	N/A	N/A	N/A	0.147301974	N/A	N/A
Carotenoid_46	zeaxanthin-laurate-palmitate	xanthophylls	989.9	533.4	988.9	positive	[M+H]+	C68H108O4	N/A	0.00253744017	N/A	N/A	N/A	N/A
Carotenoid_56	zeaxanthin	xanthophylls	569.4	477.5	568.428	positive	[M+H]+	C40H56O2	7.70536652	8.8624974	7.45402166	5.90239395	8.48678414	21.3112868
Carotenoid_57	violaxanthin	xanthophylls	601.4	221	600.4179	positive	[M+H]+	C40H56O4	100.408128	110.641831	118.694702	129.832213	140.246476	138.48582
Carotenoid_58	neoxanthin	xanthophylls	601.4	565.5	600.4179	positive	[M+H]+	C40H56O4	167.3846	178.516961	170.697602	184.844813	202.361013	201.324783
Carotenoid_59	lutein	xanthophylls	551.5	175.4	568.428	positive	[M+H-18]+	C40H56O2	1980.61443	2148.76171	2098.93658	2283.76732	2469.86784	2480.75958
Carotenoid_60	β-cryptoxanthin	xanthophylls	553.5	177.4	552.4331	positive	[M+H]+	C40H56O	9.276084	9.81063476	9.26950889	21.7427551	22.5270925	19.5817861
Carotenoid_62	8'-apo-beta-carotenal	xanthophylls	417.3	325.3	416.638	positive	[M+H]+	C30H40O	0.109914058	0.108796878	0.0839433488	0.473607728	0.454881057	0.410792136
Carotenoid_64	α-cryptoxanthin	xanthophylls	553.5	123.1	552.43	positive	[M+H]+	C40H56O	1.76341629	1.82976899	2.0100348	0.613882822	0.536427313	0.500318147
Carotenoid_67	echinenone	xanthophylls	551.6	203.1	550.9	positive	[M+H]+	C40H54O	0.0189488431	0.0211735692	0.0180477185	0.437834943	0.448581498	0.370864713
Carotenoid_21	lutein oleate	xanthophylls	815.7	533.4	832.7	positive	[M+H-18]+	C58H88O3	0.397905892	0.413952133	0.440382831	0.103244225	0.0772832599	0.1008498
Carotenoid_30	violaxanthin palmitate	xanthophylls	839.8	821.8	838.8	positive	[M+H]+	C56H86O5	0.539861948	0.469136316	0.424286543	3.25371693	3.27865639	2.49516566
Carotenoid_55	antheraxanthin	xanthophylls	585.5	175.4	584.4229	positive	[M+H]+	C40H56O3	14.8997083	17.6352758	17.5078886	16.9408862	20.4852203	23.2179306
Carotenoid_65	capsorubin	xanthophylls	601.4	109	600.42	positive	[M+H]+	C40H56O4	0.411629399	0.381694069	0.415123743	0.529363713	0.516321586	0.500581612
Carotenoid_68	β-citraurin	xanthophylls	433.3	341.1	432.6	positive	[M+H]+	C30H40O2	0.0135345324	0.0169327575	0.0126003287	0.0268565729	0.0263566079	0.0266144707
Carotenoid_54	β-cryptoxanthin oleate	xanthophylls	817.8	535.4	816.8	positive	[M+H]+	C58H88O2	N/A	N/A	N/A	N/A	N/A	N/A
Carotenoid_08	antheraxanthin dipalmitate	xanthophylls	1061	805	1060	positive	[M+H]+	C72H115O5	0.0589093914	0.0377223725	0.0533317865	1.96433431	2.09642731	1.98677702
Carotenoid_27	violaxanthin dibutyrate	xanthophylls	741.6	653.5	740.6	positive	[M+H]+	C48H68O6	N/A	N/A	N/A	0.0682872743	0.0520982379	0.0810469018
Carotenoid_29	violaxanthin myristate	xanthophylls	811.8	793.7	810.8	positive	[M+H]+	C54H82O5	N/A	N/A	N/A	0.9500084	0.906517621	0.690967614
Carotenoid_45	zeaxanthin dimyristate	xanthophylls	990	761.8	989	positive	[M+H]+	C68H108O4	0.0191840754	0.0210899063	N/A	0.0634359513	N/A	N/A

Carotenoid_09	lutein caprate	xanthophylls	705.7	533.5	722.7	positive	[M+H-18]+	C50H74O3	N/A	N/A	N/A	0.366803864	0.332491189	0.334133671
Carotenoid_15	lutein dilaurate	xanthophylls	733.5	533.3	933.5	positive	[M+H-201]+	C64H101O4	N/A	N/A	N/A	1.8101176	1.84321145	1.65377178
Carotenoid_18	lutein dipalmitate	xanthophylls	789.8	533.5	1044.8	positive	[M+H-256]+	C72H116O4	N/A	N/A	N/A	0.558345233	0.77235022	0.621713519
Carotenoid_28	violaxanthin laurate	xanthophylls	783.7	583.4	800.7	positive	[M+H-18]+	C52H80O6	N/A	N/A	N/A	0.292679546	0.298986784	0.211339448
Carotenoid_48	zeaxanthin dipalmitate	xanthophylls	789.5	533.5	1045.1	positive	[M+H-256]+	C72H116O4	N/A	N/A	N/A	0.228628727	0.232422907	0.201508463
Carotenoid_51	β-cryptoxanthin laurate	xanthophylls	735.8	535.5	734.8	positive	[M+H]+	C52H78O2	0.0400386934	0.054407076	N/A	0.196938051	N/A	N/A
Carotenoid_52	β-cryptoxanthin myristate	xanthophylls	763.9	535.5	762.9	positive	[M+H]+	C54H82O2	0.0437443127	0.0549531738	N/A	0.312217556	N/A	N/A
Carotenoid_66	canthaxanthin	xanthophylls	565.5	203.3	564.8	positive	[M+H]+	C40H52O2	N/A	N/A	N/A	0.00507664847	0.00578812775	0.0054327045
Carotenoid_20	lutein dioleate	xanthophylls	815.7	533.4	1096.7	positive	[M+H-282]+	C76H120O4	N/A	N/A	N/A	0.026150777	0.0582940529	0.0589287401
Carotenoid_53	β-cryptoxanthin palmitate	xanthophylls	791.9	535.5	790.9	positive	[M+H]+	C56H86O2	N/A	N/A	N/A	N/A	N/A	N/A
Carotenoid_17	lutein dimyristate	xanthophylls	761.8	533.5	988.8	positive	[M+H-228]+	C68H108O4	N/A	N/A	N/A	0.743572029	0.895762115	0.804280069
Carotenoid_61	astaxanthin	xanthophylls	597.3	147.1	596.84	positive	[M+H]+	C40H52O4	N/A	N/A	N/A	N/A	N/A	N/A
