# Supplementary material for: Development of cassava common mosaic virus-based vector for protein expression and gene editing in cassava
Source: Plant Methods. 2023 Aug 3;19:78. doi: 10.1186/s13007-023-01055-5 (PMC10399001; doi:10.1186/s13007-023-01055-5)
Supplement: Supplementary file 3 — Additional file 3: Table S2. Differentially accumulated carotenoids in cassava leaves infected with CsCMV2-crtB vs. CsCMV2-GFP [file 13007_2023_1055_MOESM3_ESM.pdf]

Additional file 1: Table S2 Differentially accumulated carotenoids in cassava leaves infected with CsCMV2-crtB vs. CsCMV2-GFP (log2(fold-change ≥ 2 and ≤ 0.5)

Index	Compounds	Class	CsCMV2-GFP1	CsCMV2-GFP2	CsCMV2-GFP3	CsCMV2-crtB1	CsCMV2-crtB2	CsCMV2-crtB3	Pvalue	Fold_Change	Log2FC	Type
Carotenoid_01	α-carotene	carotenes	44.2858254	60.2183143	59.2194509	7.92587148	9.25852423	9.76136505	0.011821746896064895	0.16458080757483706	-2.6031319877486876	down
Carotenoid_02	lycopene	carotenes	0	0	0	1.17029189	1.68477974	0.775609574	0.04419041672969194	Inf	Inf	up
Carotenoid_06	(E/Z)-phytoene	carotenes	3.34367101	3.95406868	4.27341454	10.0570139	12.1095374	8.39860314	0.022036504264624432	2.641495725703416	1.401355075416882	up
Carotenoid_12	lutein palmitate	xanthophylls	1.06769006	0.942971904	1.0832966	2.79836203	3.04022026	2.48833047	0.005336316446632634	2.69134592068829	1.4283278335602745	up
Carotenoid_11	lutein myristate	xanthophylls	0.0313729341	0.0294942768	0.0300003867	1.61329483	1.59199119	1.40666617	0.0018890328801113073	50.754639847548916	5.665467810368858	up
Carotenoid_63	capsanthin	xanthophylls	0	0	0	0.147301974	0	0	N/A	Inf	Inf	up
Carotenoid_60	β-cryptoxanthin	xanthophylls	9.276084	9.81063476	9.26950889	21.7427551	22.5270925	19.5817861	0.0041711829863802065	2.2517675654222646	1.1710579155535508	up
Carotenoid_62	8'-apo-beta-carotenal	xanthophylls	0.109914058	0.108796878	0.0839433488	0.473607728	0.454881057	0.410792136	6.739111556646205e-4	4.425117991919472	2.145715923915996	up
Carotenoid_64	α-cryptoxanthin	xanthophylls	1.76341629	1.82976899	2.0100348	0.613882822	0.536427313	0.500318147	7.493310135317879e-4	0.29458565939462433	-1.763240894058169	down
Carotenoid_67	echinenone	xanthophylls	0.0189488431	0.0211735692	0.0180477185	0.437834943	0.448581498	0.370864713	0.0036390101999197555	21.61386155934172	4.433884943800061	up
Carotenoid_21	lutein oleate	xanthophylls	0.397905892	0.413952133	0.440382831	0.103244225	0.0772832599	0.1008498	7.509769769695135e-5	0.22469901341407758	-2.1539343047965223	down
Carotenoid_30	violaxanthin palmitate	xanthophylls	0.539861948	0.469136316	0.424286543	3.25371693	3.27865639	2.49516566	0.009238476132734772	6.298496248554737	2.655007429611227	up
Carotenoid_08	antheraxanthin dipalmitate	xanthophylls	0.0589093914	0.0377223725	0.0533317865	1.96433431	2.09642731	1.98677702	3.240044086052573e-4	40.32672355295211	5.333664289970908	up
Carotenoid_27	violaxanthin dibutyrate	xanthophylls	0	0	0	0.0682872743	0.0520982379	0.0810469018	0.015208629851588823	Inf	Inf	up
Carotenoid_29	violaxanthin myristate	xanthophylls	0	0	0	0.9500084	0.906517621	0.690967614	0.008778290724076621	Inf	Inf	up
Carotenoid_09	lutein caprate	xanthophylls	0	0	0	0.366803864	0.332491189	0.334133671	0.0010505200447228375	Inf	Inf	up
Carotenoid_15	lutein dilaurate	xanthophylls	0	0	0	1.8101176	1.84321145	1.65377178	0.0010886848701278616	Inf	Inf	up
Carotenoid_18	lutein dipalmitate	xanthophylls	0	0	0	0.558345233	0.77235022	0.621713519	0.009376803847468645	Inf	Inf	up
Carotenoid_28	violaxanthin laurate	xanthophylls	0	0	0	0.292679546	0.298986784	0.211339448	0.010935857061789343	Inf	Inf	up
Carotenoid_48	zeaxanthin dipalmitate	xanthophylls	0	0	0	0.228628727	0.232422907	0.201508463	0.0019370250087529974	Inf	Inf	up
Carotenoid_51	β-cryptoxanthin laurate	xanthophylls	0.0400386934	0.054407076	0	0.196938051	0	0	0.6587422060495475	2.0851971692445126	1.0601838065199585	up
Carotenoid_52	β-cryptoxanthin myristate	xanthophylls	0.0437443127	0.0549531738	0	0.312217556	0	0	0.5661464353060751	3.1633789985117806	1.6614664128005616	up
Carotenoid_66	canthaxanthin	xanthophylls	0	0	0	0.00507664847	0.00578812775	0.0054327045	0.00142631354267356	Inf	Inf	up
Carotenoid_20	lutein dioleate	xanthophylls	0	0	0	0.026150777	0.0582940529	0.0589287401	0.047640056013718936	Inf	Inf	up
Carotenoid_17	lutein dimyristate	xanthophylls	0	0	0	0.743572029	0.895762115	0.804280069	0.002935837793147924	Inf	Inf	up
