# Supplementary material for: Development of cassava common mosaic virus-based vector for protein expression and gene editing in cassava
Source: Plant Methods. 2023 Aug 3;19:78. doi: 10.1186/s13007-023-01055-5 (PMC10399001; doi:10.1186/s13007-023-01055-5)
Supplement: Supplementary file 4 — Additional file 4: Table S3. Primers used for construction of the pCsCMV/1/2-NC vector [file 13007_2023_1055_MOESM4_ESM.docx]

**Table S3.** Primers used in construction of CsCMV agroinfectious clone and pCsCMV-NC vector

| Primer | Sequence (5′-3′) | |
| --- | --- | --- |
| CsCMV-5Fov | AGGAAGTTCATTTCATTTGGAGAGGGGAAAACCTCACATTCCAAACCAAAAC |  |
| NC42-R | AGGACTGGACAGAGACCACTGGGCTGTTGCAGTTGTAGGAGTGG |  |
| NC53-R | AGGACTGGACAGAGACCACTGGCAGCTTGAGTGGCTGTTGCAGT |  |
| NC-F | CAGTGGTCTCTGTCCAGTCCT |  |
| pGr35S-R | CCTCTCCAAATGAAATGAACTTCCT |  |

Underlined sequences corresponding to the overlapping region used Gibson Assembly.
