# Supplementary material for: Development of cassava common mosaic virus-based vector for protein expression and gene editing in cassava
Source: Plant Methods. 2023 Aug 3;19:78. doi: 10.1186/s13007-023-01055-5 (PMC10399001; doi:10.1186/s13007-023-01055-5)
Supplement: Supplementary file 5 — Additional file 5: Table S4. Primers used for cloning gene fragments into the pCsCMV/1/2-NC vector [file 13007_2023_1055_MOESM5_ESM.docx]

**Table S4.** Primers used for cloning of gene fragments into the pCsCMV/1/2-NC vector.

| Gene | GenBank Accession number  Phytozome Gene Identifier | Size (bp) | Primer sequence (5′-3′) |
| --- | --- | --- | --- |
| *GFP* | MK896905 | 720 | AGTGGTCTCTGTCCAGTCCTATGAGTAAAGGAGAAGAAC |
|  |  |  | GGTCTCAGCAGACCACAAGTTTATTTGTATAGTTCATCCAT |
| *crtB* | D90087 | 930 | AGTGGTCTCTGTCCAGTCCTATGAATAATCCGTCGTTAC |
|  |  |  | GGTCTCAGCAGACCACAAGTCTAGAGCGGGCGCTGCCA |
| *XopAO1* | CP083575.1 | 624 | AGTGGTCTCTGTCCAGTCCTATGCCAAGGTCCATCAGAA |
|  |  |  | GGTCTCAGCAGACCACAAGTTCATCGACCCCTCATTCG |
| *gMePDS1* | Manes.05G193700.1 | 99 | ATA*GGCCTCGTCGGCC*GATCTCTGTAAGGGACGGC |
|  |  |  | ATT*GGCCAGACTGGCCC*TAGCACCGACTCGGTGCC |
| *gMePDS2* | Manes.05G193700.1 | 99 | ATA*GGCCTCGTCGGCC*GCGTACAAAGCTTCCCAGAT |
|  |  |  | ATT*GGCCAGACTGGCC*CTAGCACCGACTCGGTGCC |

Underlined sequences corresponding to the adapter 1 and 2 sequence of Nimble Cloning. The *Sfi* I sites are marked in italics.
